# Ethnic disparities and morbidity in the Province of Antwerp, Belgium

**Published:** 2017-12

**Authors:** C Claeys, L De Souter, G Martens, E Martens, B Blauleiser, E Faes, F Caris alias Reynders, K Wuyts, Y Jacquemyn

**Affiliations:** Antwerp University Hospital UZA and Antwerp University UA – ASTARC, 2650 Edegem, Belgium; Study Centre for Perinatal Epidemiology SPE, 1060 Brussels, Belgium; AZ Sint-Jozef Malle, 2390 Malle, Belgium; Heilig Hart ziekenhuis,2500 Lier, Belgium

**Keywords:** Perinatal mortality, perinatal morbidity, obstetric outcome, social inequality, educational level, postal code

## Abstract

**Objective:**

This study aims to identify geographical disparities in perinatal mortality and morbidity in the province of Antwerp, Belgium. We performed a retrospective cohort study from an existing database. Data included from 1 January , 2000 to 31 December, 2009 and including all deliveries in the Province of Antwerp, Belgium. Collected outcome measures : fetal death, early and late neonatal death, preterm birth, low birth weight. Outcomes were analyzed according to postal code of the pregnant women’s address.

**Results:**

A total of 167.246 deliveries in sixty postal codes were analyzed and statistically significant differences (p<0.001) between postal codes for all outcome measures except for early and late neonatal death were detected. Generally postal codes tend to have either high or low prevalences for all perinatal outcomes and two postal code zones had a significantly worse perinatal outcome on all fields. Major differences in perinatal outcome exist within the well-defined area of the relatively small province of Antwerp, Belgium.

**Conclusion:**

Perinatal outcome is strongly influenced by maternal postal code even within a relatively affluent European region demonstrating persistent health inequalities and suggesting further research is necessary to explain these differences and create interventions to diminish inequalities.

## Introduction

In the Netherlands geographical differences in perinatal mortality and morbidity have been demonstrated, identification of high risk zones can have benefit from more intensive preventive care (Poeran et al., 2011; Ravelli et al., 2011a; 2011b). In Rotterdam perinatal mortality and morbidity were compared between different city areas and inequality was evident, as explanation a combination of medical, socio-economic and urban-related factors were identified . A comparable study was performed in Amsterdam (Agyemang et al., 2009). Based on risk factors including parity, maternal age, ethnicity, socio-economic status and income, it was clear that in some city areas perinatal mortality is higher and this is related to socio-economic and educational differences. Postal codes in the Netherlands have been demonstrated to be useful to identify high and low risk zones for perinatal morbidity and mortality. Our study is, as far as we know, the first that focusses on inter-city differences in Belgium. The aim of the current study was to identify geographical disparities in perinatal mortality and morbidity in the province of Antwerp, Belgium

## Material and methods

A retrospective cohort study in the Province of Antwerp, Belgium was performed. Data were retrieved from an existing database. The Study Center for Perinatal Epidemiology (SPE) collects data for every delivery in the region of Flanders, including the complete province of Antwerp. Both obstetric and neonatal data are included. These data are provided directly to the SPE by each maternity, or the midwife performing a home delivery (which is < 1% of deliveries) to the SPE and providing complete data is obligatory.

Inclusion for this retrospective cohort was from January 1st 2000 until December 31st 2009. Data were classified according to postal code in the province of Antwerp. The postal code used is the one where the mother was registered with her home address at the moment of delivery. Postal codes that demonstrated less than 1.000 deliveries in this study period were excluded. This exclusion was an obligation imposed by the ethics committee as with less deliveries in a postal code area maternal identification of the mother might become possible and we had to guarantee anonymity.

As perinatal outcomes available in the database we focused on fetal death, early neonatal death, late neonatal death, early preterm birth, late preterm birth , very low birth weight and low birth weight. Fetal death was defined as every baby born death and having a birth weight of at least 500 gram. Early neonatal death was defined as death of a live born baby with a birth weight of at least 500 gram and dying before day 28 after delivery. Late neonatal death was defined as death of a live born baby with a birth weight of at least 500 gram between day 29 and day 365 after birth. Early preterm was from 22 to 31 (included) weeks, late preterm from 32 to 36 weeks. Very low birth weight was considered as 499 to1499gram, low birth weight 1500 to 2499 grams. In univariate analysis for every of these 7 perinatal outcomes it was analyzed whether the prevalence of the outcome was different for women living in the different postal code areas and whether postal codes exist that demonstrate significantly different outcomes not only on 1 but also on different outcome measures.

The study was approved by the Antwerp University Hospital ethics committee under number13/16/174.

## Statistics

Descriptive statistics were performed for the different outcome parameters according to postal code in the province of Antwerp. As all outcome measures were dichotomic (present or absent), Chi-squared test was used, significance accepted at p<0.05. Multinomial logistic regression was planned for those outcome measures that showed significant differences according to postal code including socioeconomic factors as cofactors. All calculations were performed with SPSS 22.

## Results

In the study period a total number of 206970 deliveries in the province of Antwerp was registered. In total 62 different postal codes were retained with more than 1000 deliveries in the period studied, representing 167246 deliveries; i.e. 80.8% of deliveries in Antwerp and 38 postal codes were excluded because less than 1000 deliveries were noted in the study period.

**Figure 1:**
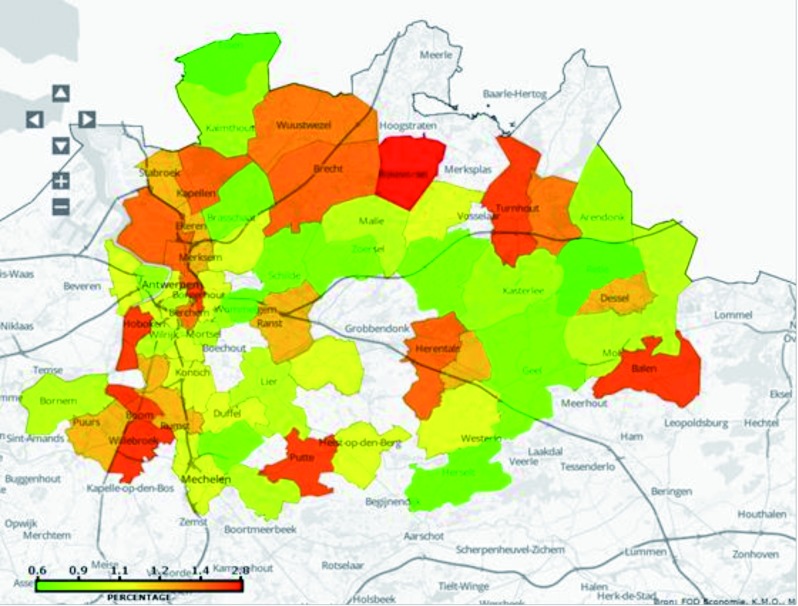
— Early preterm birth: 22 to 31 weeks

For descriptive purposes the ratio of the outcome measure over the total number of deliveries for a given postal code was used. Figure 1 demonstrates this for the outcome “Early Preterm Birth” on a map of the Province of Antwerp.

For fetal death, early and late preterm birth, very low birthweight and low birthweight differences between postal codes were significant (all parameters: p< 0.001), but for early neonatal death (p= 0.208) and for late perinatal death (p= 0.235) differences were not statistically significant.

From the data it became clear that there existed postal codes with a high prevalence of bad obstetrical outcomes (scoring “present” on all or most perinatal outcomes) and postal codes that performed better on all or most outcomes. Table I presents the postal codes that are in the highest and those that are in the lowest quintile for at least 5 of the 7 perinatal outcome measures we selected. From Table I it becomes clear that there exist postal codes with a generally worse and others with a generally better perinatal outcome.

**Table I:** Postal codes that are in the highest and those that are in the lowest quintile for the prevalence of at least 5 of 7 perinatal outcome measures.

≤P5		≥P95	
Postal Code	City/ village Name	Postal Code	City/ village Name
2018	Antwerpen 1	2030	Antwerpen 3
2275^1^	Gierle – Lille – Poederlee –Wechelderzande	2310^2^	Rijkevorsel
2370	Arendonk	2830^2^	Blaasveld – Heindonk – Tisselt – Willebroek
2460	Kasterlee – Lichtaart – Tielen	2845	Niel
2820	Bonheiden – Rijmenam	2940	Hoevenen – Stabroek
2860	Sint Katelijne Waver	2950	Kapellen
2920^1^	Kalmthout		
2970	’s Gravenwezel – Schilde		
2980	Halle – Zoersel		

^1^postal codes 2275 and 2920 are in the lowest quintile for each of 7 outcome measures, so show best perinatal outcomes in general

^2^ postal codes 2310 and 2830 are in the highest quintile for each of 7 outcome measures, so show worst perinatal outcomes in general

## Discussion

The strength of this study is that a complete data set over a period of 10 years in a relatively affluent European province can be studied. Also our data are not limited to perinatal mortality but do include preterm birth and low birth weight. It is clear that there exist major differences in perinatal outcome in the Province of Antwerp depending on where the pregnant woman lives. Postal code as an approximation for home address is closely related to such socio economic factors as income and education as people with a comparable income, often also ethnic and religious background tend to live in the same area.

At first we hypothesized that suboptimal perinatal outcome would be higher in the city, with its higher burden of socioeconomic fugitives , as compared to more rural areas of the province. As can be noted for instance in Figure 1, there are both rural and city areas of high risk and bad outcome postal codes are distributed all over the province of Antwerp. Data on migrant population can be easily found at the Flemish Department of Statistics (http://www.statistiekvlaanderen.be/monitor-lokale-inburgering-en-integratie). Clearly there is no link between the percentage of allochthonous inhabitants (and mothers giving birth), up to 60 % in the Antwerp inner city (postal code 2030) and less than 10 % e.g. in Rijkevorsel and Balen (postal codes 2310 and 2830), but all these scored in the highest risk postal codes for all 7 outcome measures. The relation with migrants then cannot be sustained by our data.

Weaknesses include that the available data set does not provide data that might be relevant such as maternal professional work, marital status, recent migration, ethnicity and other factors influences perinatal outcome. We have previously reported that ethnicity in Flanders does influence perinatal outcome (Jacquemyn et al., 2012a).We did try to perform a more detailed analysis as part of the original set-up of this study as SPE data are linked to results of a questionnaire on socioeconomic factors (including maternal educational level, nationality, marital status and actual profession) filled in independently at the moment the newborn is registered at the community civil department, but it is not obligatory to fill in all question fields. It became evident that these data were very incomplete (23.9 % complete) except for maternal educational level, for which 99.98% was complete. Actually we did test whether for each of the 7 outcome measures maternal educational level (expressed as finished primary school versus left school before finishing primary school) was significantly different between those demonstrating the outcome (fetal death, early and late neonatal death, early and late preterm birth, very low and low birthweight). This was the case for all (all p < 0.001) except for early neonatal death ( p=0.08). Maternal education has been shown to be an important socio-demographic factor related to perinatal outcome in Flanders before (Cammu et al., 2011; Jacquemyn, 2012b).

As a very crude proxy for socio economic status we did try to look at mean income data such as available at governmental websites (https://statbel.fgov.be/nl/themas/huishoudens/fiscale-inkomens#figures), but the data as available are not per postal code number, they are only publicly available per complete city, the city of Antwerp by itself consists of 6 different postal codes, as can be appreciated from Table I, these postal codes are part both of the ones with the best and those with the worst perinatal outcome. This made it impossible to link the more detailed postal code data to financial income data. The Belgium Privacy Committee did not allow us to get access to more detailed data. It is possible to get a view on the mean fiscal income (for the year 2014 as found on https://statbel.fgov.be/nl/themas/huishoudens/fiscale-inkomens#figures) for the smaller areas as mentioned in Table I, we demonstrate this in Table II. It is clear from this table there is no simple division relating high or low income with perinatal outcome, actually the postal code with the worst outcome (2830) has almost the same income (Euro 50753) as the one with the very best outcome (2275, Euro 50477)

**Table II:** Financial mean yearly income income for postal codes that are in the highest and those that are in the lowest quintile for the prevalence of at least 5 of 7 perinatal outcome measures.

≤P5			≥P95		
Postal Code	City/ village Name	Euro	Postal Code	City/ village Name	Euro
2018	Antwerpen 1	Not aivalable	2030	Antwerpen 3	Not Available
2275	Gierle – Lille – Poederlee – Wechelderzande	50477	2310	Rijkevorsel	47390
2370	Arendonk	46928	2830	Blaasveld – Heindonk – Tisselt – Willebroek	50753
2460	Kasterlee – Lichtaart – Tielen	53710	2845	Niel	48605
2820	Bonheiden – Rijmenam	67843	2940	Hoevenen – Stabroek	54930
2860	Sint Katelijne Waver	57938	2950	Kapellen	61877

Financial data as available on https://statbel.fgov.be/nl/themas/huishoudens/fiscale-inkomens#figures (accessed 26 March, 2018).

The exclusion of postal code with less than 1.000 deliveries in the period studied might mask some influence, this exclusion was obliged by the ethical committee to guarantee maternal anonymity as otherwise in a small group individuals could be identified. Of course this limitation means that for very small villages in the province the study provides no information. When comparing our data with those from the city of Rotterdam it was kept in mind that the geographic region covered by a postal code in the Netherlands is much smaller than in Belgium. In Belgium this generally includes a complete city or village, not an area of a city or village. The available database does not include the complete address of women, the postal code is the most detailed information available. We analyzed data according to the postal code where the mother lives as a proxy for socio-economic status or living situation. It could be suggested that for some postal codes the distance to maternity might be longer and this can influence obstetric outcome (Ravelli et al., 2011b). But specifically for the region of Flanders we have demonstrated in the past that due to the fact that even in the small regions there are several easily reached maternities, distance and even place of birth do not influence perinatal outcome (Hauspy et al., 2001).

The fact that a few postal codes were in the highest quintiles for demonstrating complicated outcomes and others in the lower quintiles should not be completely related to postal, code, as it is evident that some of our outcome measures are by themselves closely related such as preterm birth and low birth weight.

The difficulties encountered while performing this research demonstrate the need for more high quality and complete registration of data. Another problem hampering further analysis was that due to limitations imposed by the Ethics Committee, based on obligatory privacy concerns as imposed in Belgium linkage with data such as financial status was not possible.

## Conclusion

This study demonstrates that perinatal outcome is unequally distributed in the relatively affluent Province of Antwerp, Belgium. Some postal codes can be considered as high risk regions for a bad obstetrical outcome. There was no simple relationship with the ratio of migrants nor with family income in the different areas but the available data are to general to definitively exclude such a relationship. In addition to postal code maternal educational level and many other factors also influence maternal outcome. Further research is necessary to analyze factors contributing to these inequalities and to develop strategies that can help minimize perinatal inequality. Alternative ways or interpretations should be studied to solve issues resulting in limitations on studies due to privacy regulations.

## Conflict of interest

None of the authors has a conflict of interest.

## References

[1] Agyemang C, Vrijkotte TG, Droomers M (2009). The effect of neighbourhood income and deprivation on pregnancy outcomes in Amsterdam, The Netherlands.. J Epidemiol Community Health.

[2] Cammu H, Martens G, Keirse MJ (2011). Mothers’ level of education and childbirth interventions: A population-based study in Flanders, Northern Belgium.. Birth.

[3] Hauspy J, Jacquemyn Y, Van Reempst P (2001). Intrauterine versus postnatal transport of the preterm infant: a short distance experience.. Early Human Development.

[4] Jacquemyn Y, Benjahia N, Martens G (2012a). Pregnancy outcome of Moroccan and Turkish women in Belgium. Clin Exp Obst Gyn.

[5] Jacquemyn Y (2012b). Ethnic disparities: genetics vs (social) environment. World J Obstet Gynecol.

[6] Poeran J, Denktas S, Birnie E (2011). Urban perinatal health inequalities.. J Matern Fetal Neonatal Med.

[7] Ravelli AC, Steegers EA, Rijninks-van Driel GC (2011a). Differences in perinatal mortality between districts of Amsterdam.. Ned Tijdschr Geneeskd.

[8] Ravelli AC, Rijninks-van Driel GC, Erwich JJ (2011b). Differences between Dutch provinces in perinatal mortality and travel time to hospital.. Ned Tijdschr Geneeskd.

